# Impact of Citrate and Lipid-Functionalized Magnetic Nanoparticles in Dehydropeptide Supramolecular Magnetogels: Properties, Design and Drug Release

**DOI:** 10.3390/nano11010016

**Published:** 2020-12-23

**Authors:** Sérgio R. S. Veloso, Joana F. G. Silva, Loic Hilliou, Cacilda Moura, Paulo J. G. Coutinho, José A. Martins, Martín Testa-Anta, Verónica Salgueiriño, Miguel A. Correa-Duarte, Paula M. T. Ferreira, Elisabete M. S. Castanheira

**Affiliations:** 1 Centro de Física (CFUM), University of Minho, Campus de Gualtar, 4710-057 Braga, Portugal; sergioveloso96@gmail.com (S.R.S.V.); joanasilva.fg@gmail.com (J.F.G.S.); cmoura@fisica.uminho.pt (C.M.); pcoutinho@fisica.uminho.pt (P.J.G.C.); 2 Institute for Polymers and Composites, Department of Polymer Engineering, University of Minho, Campus de Azurém, 4800-058 Guimarães, Portugal; loic@dep.uminho.pt; 3 Centro de Química (CQUM), University of Minho, Campus de Gualtar, 4710-057 Braga, Portugal; jmartins@quimica.uminho.pt (J.A.M.); pmf@quimica.uminho.pt (P.M.T.F.); 4 Departamento de Física Aplicada, Universidade de Vigo, 36310 Vigo, Spain; mtesta@uvigo.es (M.T.-A.); vsalgue@uvigo.es (V.S.); 5 CINBIO, Universidade de Vigo, 36310 Vigo, Spain; macorrea@uvigo.es

**Keywords:** magnetic gels, drug release, magnetic hyperthermia, magnetic lipogels, supramolecular hydrogels, magnetic nanoparticles, self-assembly, nanoparticle functionalization

## Abstract

Currently, the nanoparticle functionalization effect on supramolecular peptide-based hydrogels remains undescribed, but is expected to affect the hydrogels’ self-assembly and final magnetic gel properties. Herein, two different functionalized nanoparticles: citrate-stabilized (14.4 ± 2.6 nm) and lipid-coated (8.9 ± 2.1 nm) magnetic nanoparticles, were used for the formation of dehydropeptide-based supramolecular magnetogels consisting of the ultra-short hydrogelator Cbz-L-Met-*Z*-ΔPhe-OH, with an assessment of their effect over gel properties. The lipid-coated nanoparticles were distributed along the hydrogel fibers, while citrate-stabilized nanoparticles were aggregated upon gelation, which resulted into a heating efficiency improvement and decrease, respectively. Further, the lipid-coated nanoparticles did not affect drug encapsulation and displayed improved drug release reproducibility compared to citrate-stabilized nanoparticles, despite the latter attaining a stronger AMF-trigger. This report points out that adsorption of nanoparticles to hydrogel fibers, which display domains that improve or do not affect drug encapsulation, can be explored as a means to optimize the development of supramolecular magnetogels to advance theranostic applications.

## 1. Introduction

Supramolecular magnetogels basically comprise two main components: the hydrogel and the magnetic nanoparticles. Following a stimulus, the self-assembly of the supramolecular hydrogelators is driven towards a kinetically-trapped intertwined fibrillar structure, such that solvent pocket microdomains are formed. This process takes place through the cooperative effect of different non-covalent intermolecular interactions: hydrogen bonding, van der Waals, electrostatic, and/or hydrophobic and aromatic interactions [1,2,3,4,5,6,7].

For example, dehydropeptide-based hydrogelators, such as the minimalist Cbz-L-Met-*Z*-ΔPhe-OH (see Structure S1 in Supplementary Materials), have been used due to the simplicity of producing biocompatible systems at low cost, and its promising properties for drug delivery [8,9,10]. The aforementioned dehydropeptide uses of a non-polar amino acid, methionine, to induce hydrophobic collapse, while the dehydrophenylalanine moiety provides a means for π-π interactions (like the *N*-capping group) and conformational constraints in the peptide backbone, meaning that it promotes the self-assembly into fibers and provides resistance to enzymatic degradation [8,9,10]. As assessed in this work, the hydrogel displays a moderate gelation kinetics and elastic modulus, which allows following the impact of the nanoparticles, so that diverse parameters can be optimized. Further, the gels can be formed in a skin pH range of 4-6 [11], thus being suitable for dermal application.

The retention or entrapment of nanoparticles during the gelation process allows for the tailoring and modulation of the matrix structure, along with the possibility of tuning different physical properties [3,12,13]. The modulation of the matrix structure also enables optimizing the loading of a wide variety of hydrophilic and hydrophobic drugs, reducing potential side effects, and enabling higher doses in therapy at the right location when using magnetogels for drug delivery. In this regard, taking advantage of the magnetic nanoparticles, the application of a magnetic field gradient allows the control and targeting of the nanosystem to a specific location [14,15,16], which can be further coupled with an alternating magnetic field (AMF), such that the nanoparticles can absorb energy and then release it as heat [17,18,19,20]. As a consequence, there is a synergistic effect that involves this magnetic hyperthermia and the subsequently associated enhancement of drug release [21,22,23], such that a much higher therapeutic efficiency can be attained. Along these lines, among transition metal ferrites, manganese ferrite nanoparticles exhibit very suitable magnetic properties, particularly in terms of the high magnetic susceptibility and in terms of magnetophoretic mobility, which render them as an appealing option to improve the supramolecular magnetogels for drug delivery applications [24,25]. Further, manganese ferrites display good biocompatibility and the large saturation magnetization reduces the required concentration of nanoparticles, thus averting side effects [8,25].

Supramolecular magnetogels endorsed with bare nanoparticles were demonstrated to be easily prone to aggregation, which led us to limit the concentration of nanoparticles that were encapsulated [8,9]. Alternatively, the use of a thick shell coating the nanoparticles was confirmed to hamper the gelation process, with the additional cost of requiring a larger concentration of hydrogelator to compensate for the bulkier nanocomposite [10]. On the other hand, forcing the supramolecular design strategies to conjugate the nanoparticles with the hydrogelator molecules has also been considered [25,26,27]. In general, these existing strategies for supramolecular magnetogels either imply complex fabrication steps and/or fail on the homogeneous incorporation of high concentrations of magnetic material and target drugs, which strongly limit their usability in theranostics. Furthermore, the current literature on supramolecular magnetogels lacks exploration of the composite functionalization and its effect on supramolecular magnetic gels properties. Consequently, the large amounts of hydrogelator employed and the aggregation of nanoparticles in most of the reported systems have demonstrated lower heating efficiency, hindering the sought on-demand controlled release of the payload.

Doxorubicin is one of the most commonly used chemotherapeutic drugs in a wide variety of cancers. However, its various side effects (e.g., neutropenia and heart failures) lead to a worsening of the patient’s quality of life, which can be improved through incorporation with drug delivery system to increase the safety profile of the therapy. Considering this need, a useful strategy could be the encapsulation of doxorubicin in liposomal systems, such as the liposomal formulation Doxil^®^, or the use of citrate-stabilized nanoparticles, which strongly adsorb the positively charged drug molecules through electrostatic interactions [28].

Herein, supramolecular magnetogels endorsed with high nanoparticle concentration, employing a lipid coating (magnetoliposome-like structure) or citrate stabilization, are evaluated. Whereas the citrate stabilization provides electrostatic stabilization, the lipid coating assessment tackles the strategy, not only to ensure the steric stabilization of manganese ferrite nanoparticles, but also to provide the structure with enough hydrophobic cavities for an efficient drug loading and subsequent controlled drug diffusion. This option to obtain magnetogels systems, compared with the use of negatively-charged citrate-stabilized nanoparticles, demonstrates that the gelation becomes optimized, such that the concentration of stable magnetic nanoparticles within the gel is increased. As a consequence, besides improving the system stability and magnetic properties, the strategy demonstrates the role of the magnetic nanoparticles taking advantage of the hyperthermia they are responsible for, on drug release, using the antitumor drug doxorubicin.

## 2. Materials and Methods

### 2.1. Synthesis Procedure of Magnetic Nanoparticles

#### 2.1.1. Preparation of Citrate-Stabilized Manganese Ferrite Nanoparticles

A modified synthesis of citrate-stabilized nanoparticles was adapted from reference [29]. Trisodium citrate dehydrate (1 mmol) and NaOH (4 mmol) were added to 19 mL of ultrapure water at 100 °C. A 1 mL aqueous solution of FeSO_4_·7H_2_O (1.33 mmol) and MnSO_4_·H_2_O (all reagents from Merck-Sigma, St. Louis, MO, USA) (0.66 mmol) was added, drop by drop, into the mixture under vigorous agitation. After 2 h, the solution was cooled down to room temperature, washed through magnetic decantation with water/ethanol 1:1, and dried at 100 °C.

#### 2.1.2. Preparation of Lipid-Coated Manganese Ferrite Nanoparticles

Lauric acid (1 mmol) and NaOH (4 mmol) were added to 19 mL of ultrapure water at 100 °C. A 1 mL aqueous solution of FeSO_4_·7H_2_O (1.33 mmol) and MnSO_4_·H_2_O (0.66 mmol) was added, drop by drop, into the mixture under vigorous agitation. After 2 h, the solution was cooled down to room temperature, washed through repeated centrifugation with water, and dried at 100 °C. The stock solution was prepared by dispersion of the nanoparticles (4 mg) in 2 mL of 2 mM L-α-phosphatidylcholine (from egg yolk, egg-PC) (Merck-Sigma, St. Louis, MO, USA) solution through sonication at 190 W. The lipid-coated nanoparticles were then washed and purified with ultrapure water by magnetic decantation.

### 2.2. Self-Assembly of Magnetogels

#### 2.2.1. Optimization of Hydrogel Gelation

Gelation optimization was carried out through turbidity measurements at 500 nm. The hydrogel and glucono-δ-lactone (GdL) concentrations were screened. The self-assembly was induced by dissolving the hydrogelator in basic pH through the addition of 2 v/v% NaOH (1 M) and, then, glucono-δ-lactone (GdL) was added to decrease the pH homogeneously. A solution of 0.05 wt% hydrogel was used to assess the fiber-catalyzed secondary nucleation. The aggregates fraction, f(t), was defined as follows:

(1) $$f\left(t\right)=\frac{T_{obs}-T_{free}}{T_{agg}-T_{free}}$$

where $T_{t}$, $T_{free}$ and $T_{\infty }$ stand for turbidity measured at 500 nm observed at time t, before the addition of GdL and when aggregated (turbidity at t=5 h), respectively. An empirical exponential decay function was fitted according to Equation (2) [30]:

(2) $$T\left(t\right)=\frac{T\left(\infty \right)}{\sqrt[{v}]{\left(1+ve^{-k_{emp}\left(t-t_{m}\right)}\right)}}$$

where $k_{emp}$ is the rate constant (inverse of the relaxation time) of fibril formation and $t_{m}$ is the point of the maximum elongation rate. This constant rate includes various steps and its interpretation between different systems is misleading. As a result, other models were fitted to understand the influence of the parameters on the nucleation and elongation rates. Saitô’s fractional aggregation model has been successful in the aggregation studies of β-amyloid, calcitonin, prion, and α-sinuclein [31,32,33]. At a concentration larger than critical micellar concentration ([M]≫cmc), the aggregation can be described by a two-step mechanism scheme [33]:

$$n_{0}M\left(monomers\right)⇌M_{n_{0}}\left(micelle\right)\overset{k_{n}}{\to }\;P_{n}\;M+P_{n}\overset{k_{e}}{\to }\;P_{n+1}.$$

In this mechanism, $k_{n}$ and $k_{e}$ represent the average nucleation and growth rate constants, M is the monomer, $M_{n_{0}}$ represents the micelle (precatalytic form of the monomer), $P_{n}$ is the nucleus of fibril with n monomer molecules, and $P_{n+1}$ is the extended fibril with n+1 monomer molecules; thus, P is both a product and catalyst in the growth step reaction. The fibril formation can be described according to the equation:

(3) $$f\left(t\right)=\frac{\rho \left(e^{\left(1+\rho \right)k_{s}t}-1\right)}{1+\rho e^{\left(1+\rho \right)k_{s}t}}$$

where $k_{s}=\;k_{e}\left[M\right]$ is the effective growth rate constant, $\rho =\frac{k_{n}}{k_{s}}$, and the initial condition is f(0)=0. The secondary nucleation was evaluated through the aggregation models of Knowles et al. [30,34] and Cohen et al. [35]. The former model describes the concentration of monomer in the fibers according to the equation:

(4) $$f\left(t\right)=1-e^{-C_{+}e^{kt}+C_{-}e^{-kt}+k_{n}m_{tot}^{n_{c}-1}k_{-}^{-1}}$$

where $m_{tot}$ is the total hydrogelator concentration and $k\;=\sqrt{2m_{tot}k_{+}k_{-}}$. In the absence of fibrils, at the beginning of the aggregation,

(5) $$C_{\pm }=\frac{\pm k_{n}m_{tot}^{n_{c}-1}}{2k_{-}}$$

where $k_{+}$ is the polymerization rate and $k_{-}$ is the secondary nucleation (fragmentation). The latter model describes the concentration of monomer in the fibers according to the equation:

(6) $$f\left(t\right)=1-{\left(\frac{B_{+}+C_{+}}{B_{+}+C_{+}e^{\kappa t}}\frac{B_{-}+C_{+}e^{\kappa t}}{B_{-}+C_{+}}\right)}^{\frac{k_{\infty }{}^{2}}{\kappa {ќ}_{\infty }}}-e^{-k_{\infty }t}$$

where $\kappa =\sqrt{2k_{+}k_{2}m{\left(0\right)}^{n_{2}+1}}$ is associated with the secondary pathways, $k_{2}$ is the fibril-catalyzed secondary nucleation, and $k_{2}=k_{-}$ when $n_{2}\;=\;0$, $C_{\pm }=\pm \lambda^{2}/\left(2\kappa^{2}\right)$, $\lambda =\sqrt{2k_{+}k_{n}m{\left(0\right)}^{n_{c}}}$ is related with the rate of formation of new aggregates through primary pathways, $B_{\pm }=\left(k_{\infty }\pm {ќ}_{\infty }\right)/\left(2\kappa \right)$, $k_{\infty }=\sqrt{2\kappa^{2}/\left[n_{2}\left(n_{2}+1\right)\right]+2\lambda^{2}/n_{c}}$, and ${ќ}_{\infty }=\sqrt{k_{\infty }^{2}-4C_{+}C_{-}\kappa^{2}}$. The parameters $n_{c}$ and $n_{2}$ describe the dependencies of the primary and secondary pathways, and m(0) is the initial concentration of soluble monomers.

#### 2.2.2. Development of Magnetogels

The prepared nanoparticles were added to the hydrogel solution at a final volume of 200 µL and at the required concentration from a starting solution at 2 wt%. All hydrogel/magnetogel solutions were left standing at room temperature until the gel phase was attained. Here, the unit wt% stands for m/v%.

### 2.3. Spectroscopic Measurements

#### 2.3.1. General Methods

Fluorescence measurements were carried out using a Horiba-Jobin Yvon Fluorolog 3 spectrofluorimeter (HORIBA Jobin Yvon IBH Ltd., Glasgow, UK), equipped with double excitation and emission monochromators, Glan-Thompson polarizers (HORIBA Jobin Yvon IBH Ltd., Glasgow, UK), and a temperature-controlled cuvette holder. Fluorescence emission spectra were corrected for the instrumental response of the system. The excitation of the hydrogelator was set at 280 nm, and the emission spectrum was collected between 290 nm and 600 nm with a slit of 6 nm in both excitation and emission. Absorption spectra were recorded in a Shimadzu UV-3600 Plus UV-Vis-NIR spectrophotometer (Shimadzu Corporation, Kyoto, Japan).

The fluorescence quantum yield, $\;\Phi_{s}$, can be determined by Equation (7) (standard method) [36,37],

(7) $$\Phi_{s}=\frac{\left(A_{r}F_{s}n_{s}^{2}\right)}{\left(A_{s}F_{r}n_{r}^{2}\right)}\Phi_{r}$$

where *A* is the absorbance at the excitation wavelength, *F* is the integrated emission area, and *n* is the refraction index of the solvents. Subscripts *r* and *s* refer to the reference and sample compound, respectively. The absorbance value at excitation wavelength was always less than 0.1 in order to avoid inner filter effects. L-Tryptophan in aqueous buffer solution (pH = 7.2) was used as a reference ($\Phi_{r}$ = 0.14 at 25 °C) [38].

#### 2.3.2. Fluorescence Anisotropy Measurements

The steady-state fluorescence anisotropy values, *r*, provide information on the average microviscosity of the gel matrix where the fluorophore is localized and can be determined by Equation (8) [39],

(8) $$r=\frac{I_{\mathrm{VV}}-GI_{\mathrm{VH}}}{I_{\mathrm{VV}}+2GI_{\mathrm{VH}}}$$

where I_VV_ and I_VH_ are the intensities of the emission spectra obtained with vertical and horizontal polarization, respectively (for vertically polarized excitation light), I_HV_ and I_HH_ are the emission intensities obtained with vertical and horizontal polarization (for horizontally polarized excitation light), and $G=I_{\mathrm{HV}}/I_{\mathrm{HH}}$ is the instrumental correction factor.

#### 2.3.3. FRET Measurements

The drug incorporation into the magnetogels network was investigated by Förster Resonance Energy Transfer (FRET). FRET efficiency, *Φ_FRET_*, defined as the proportion of donor molecules that have transferred their excess energy to acceptor molecules, can be expressed by Equation (9) [39],

(9) $$\Phi_{FRET}=1-\frac{I_{DA}}{I_{D}}$$

where $I_{DA}$ and $I_{D}$ are the donor integrated fluorescence intensities in the presence and absence of an acceptor, respectively. FRET efficiency can also be determined using the donor-acceptor intermolecular distance, $R_{DA},$ and the Förster radius (critical diameter), $R_{0}$, through Equation (10) [39],

(10) $$\Phi_{FRET}=\frac{1}{1+{\left(\frac{R_{DA}}{R_{0}}\right)}^{6}}.$$

The Förster radius,$\;R_{0}$, the distance at which FRET efficiency is 50%, can be determined by the spectral overlap, J(λ) between the donor fluorescence emission and the acceptor absorption, according to Equations (11) and (12) (with $R_{0}\;$ in Å, λ in nm, $\varepsilon_{A}\left(\lambda \right)$ in M^−1^ cm^−1^) [39],

(11) $$R_{0}=0.2108\;\times \;{\left[\kappa^{2}\Phi_{D}n^{-4}J\left(\lambda \right)\right]}^{1/6}$$

(12) $$J\left(\lambda \right)=\int_{0}^{\infty }I_{D}\left(\lambda \right)\varepsilon_{A}\left(\lambda \right)\lambda^{4}d\lambda$$

where $\kappa^{2}\;$= 2/3 is the orientational factor assuming random orientation of the dyes, $\Phi_{D}$ is the donor fluorescence quantum yield in the absence of energy transfer, n is the refraction index of the medium, $I_{D}\left(\lambda \right)$ is the fluorescence spectrum of the donor normalized so that $\int_{0}^{\infty }I_{D}\left(\lambda \right)\;d\lambda =1$, and $\varepsilon_{A}\left(\lambda \right)$ is the molar absorption coefficient of the acceptor.

### 2.4. Characterization Techniques

#### 2.4.1. Scanning Transmission Electron Microscopy (STEM)

STEM images were recorded using a NanoSEM—FEI Nova 200 (FEI Company, Hillsboro, OR, USA), operating at 15 kV, coupled to an Electron Dispersive Spectroscopic analyzer (EDS) and Electron Backscatter Diffraction EDAX—Pegasus X4M analyser (AMETEK Inc., Berwyn, PA, US) and detection system (EBSD) at SEMAT (Serviços de Caracterização de Materiais, Guimarães, Portugal). After preparation of the hydrogel, a small portion of each sample was placed onto a TEM 400 mesh copper grid with Formvar/Carbon (ref. S162-4 from Agar Scientific), held by tweezers and the excess solution was cleaned. The processing of STEM images was performed using ImageJ software (National Institutes of Health (NIH), version 1.52p, Bethesda, MD, USA),which consisted of enhancing local contrast and adjusting brightness followed by a manual selection of fibers.

#### 2.4.2. X-ray Diffraction

A conventional PAN’alytical X’Pert PRO diffractometer (Malvern Panalytical Ltd., Malvern, UK) was used for X-ray diffraction (XRD) analyses, operating with Cu K_α_ radiation, in a Bragg-Brentano configuration.

#### 2.4.3. Raman Spectroscopic Measurements

Raman spectroscopy was used to assess the effect of nanoparticles in the secondary structure of the hydrogel fibers. Measurements were performed at room temperature with a Jobin Yvon T64000 triple Raman Spectrometer (HORIBA Jobin Yvon IBH Ltd., Glasgow, UK), equipped with a liquid nitrogen cooled charge couple device (CCD) detector, with a resolution better than 1 cm^−1^. The excitation line, 514.5 nm, of an argon ion laser was focused onto the sample using a ×50 objective (focused to ~1.5 µm of diameter) of an Olympus Microscope BHSM (Olympus Corporation, Tokyo, Japan) in a backscattering geometry. The spectra were acquired with a measured power of about 350 µW on the sample, with a spectral acquisition time of 45 s averaged over 10 scans, over the range 770–1800 cm^−1^.

#### 2.4.4. Magnetic Properties

Magnetic measurements were performed using a SQUID magnetometer from Quantum Design (Quantum Design Inc., San Diego, CA, USA). The magnetization dependence with temperature in zero-field-cooling (ZFC) and field-cooling (FC) conditions was performed at 100 Oe in the 10–320 K range. Hysteresis loops were measured at different temperatures up to an external field of 50 kOe.

#### 2.4.5. Rheology

The viscoelastic characterization of gels was performed with a stress-controlled rotational rheometer Anton Paar MCR300 (Anton Paar GmbH, Graz, Austria). Liquid samples were loaded into the Couette geometry of the rheometer and temperature was kept at 25 °C during testing. After a five hour rest period ensuring gel setting and structural equilibrium of samples, a sweep in the strain amplitude was performed from 0.001% to 500% to assess the linear regime of viscoelasticity and the large amplitude oscillatory strain (LAOS) regime.

### 2.5. Drug Release Assays

#### 2.5.1. Incorporation of Doxorubicin

To study the incorporation and microenvironment of doxorubicin in gels through fluorescence spectroscopy, the drug was added to gel solutions prior to gelation, for a final concentration of 20 µM (to guarantee that fluorescence intensity is proportional to concentration). From the hydrogelator solution, 200 µL were transferred to a fluorescence microcuvette and left standing until the gel was formed.

#### 2.5.2. Drug Release to pH = 7 Buffer

To assess doxorubicin release through fluorescence spectroscopy, gels (100 µL) loaded with 0.1 mM doxorubicin were prepared and left stabilizing overnight in Amicon^®^ Ultra-0.5 mL centrifugal filters (MilliporeSigma, St. Louis, MO, USA) with a 0.1 µm pore size. Then, the filter tube was immersed in pH = 7 buffer (800 µL) to keep pH constant (besides neutralizing the gels), and left standing at room temperature, with or without an alternating magnetic field (AMF). The AMF was generated in a custom-designed solenoid device (800 turns per meter, length: 31 cm and internal diameter: 4.8 cm) by applying an alternating electric current. A magnetic field of 2.98 mT at 1000 kHz was used. Aliquots were taken and replaced with pH = 7 buffer, then fluorescence was measured to determine the concentration at each time point. Release profile assays were performed in triplicate.

## 3. Results

### 3.1. Optimization of Hydrogel Gelation Kinetics

**Figure 1:**
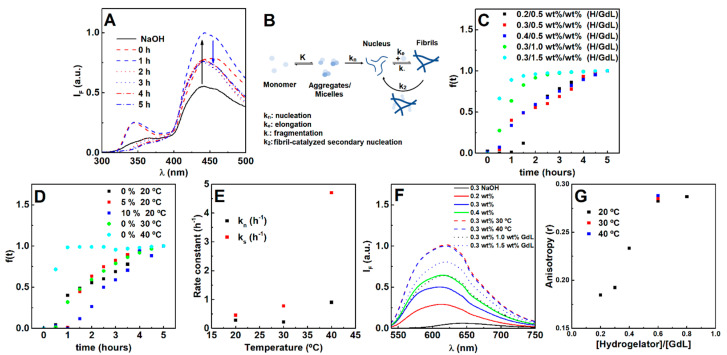
(**A**) Fluorescence emission spectra of the hydrogelator (0.3 wt%) over time after the gelation trigger (0.5 wt%). (**B**) Scheme of the evaluated pathways during the gelation process, where the fibril-catalyzed secondary nucleation contribution was found to be negligible. (**C**) Gelation kinetic profile dependence on hydrogelator and GdL concentration, (**D**) fibril concentration and temperature. (**E**) Average nucleation and elongation rate constants obtained from the Saitô’s aggregation model fitting to turbidity profiles at increasing temperatures (hydrogel 0.3 wt%, GdL 0.5 wt%). Fluorescence emission (**F**) and anisotropy *r* (**G**) of Nile Red (2 µM) in hydrogels prepared at different hydrogelator-to-GdL ratios and temperatures (fixed hydrogel and GdL concentration).

Turbidity kinetic assays were carried out to optimize hydrogel gelation, which is required to ensure quasi-homogeneous encapsulation of nanocomposites. Initially, the hydrogelator molecules were majorly organized in a mixture of micelles/aggregates and free monomer as suggested by fluorescence emissions at 450 nm and 360 nm, respectively (Figure 1A).

Once gelation is initiated (after the addition of GdL), both emission bands increase, indicating the occurrence of a reorganization process. Here, the models of Knowles et al. [34] and Cohen et al. [35] (commonly used for β-amyloid aggregation) were also used as a strategy to assess the possible occurrence and influence of monomer independent (k_-_; fragmentation) and/or monomer dependent (k_2_; fibril-catalyzed secondary nucleation) secondary pathways (Figure 1B).

The gelation is characterized by a sigmoidal profile, which is a common feature of fibrillation processes comprising a nucleation and elongation phase (Figure 1C,D) [30]. Increasing the hydrogelator concentration at the same GdL (0.5 wt%) concentration resulted in an increase of the nucleation rate, while the elongation rate decreased. The increase in GdL concentration promoted both the nucleation and elongation phases. Hereby, the primary nucleation process is dependent on both the hydrogelator and GdL, while the elongation process is majorly affected by the GdL. The elongation phase dependence on GdL concentration (for a fixed hydrogelator concentration) demonstrates that its rate can be increased if more protons are made available over time, considering that GdL proton dissociation is also a kinetically-dependent process [40]. Such results evidence that an increase of hydrogelator concentration has to be accompanied by an increase of GdL concentration to keep the molar equivalents, thus favoring both nucleation and elongation. Notably, by increasing both hydrogelator and GdL concentrations, no major differences were obtained for the final pH, while increasing GdL alone strongly decreased pH (Table S1 in Supplementary Materials).

A weak scaling of the half-time with the initial monomer concentration was obtained (−0.63), which is characteristic of monomer independent secondary pathways (such as fragmentation). Yet, the light scattering kinetic profiles cannot be satisfactorily matched using fixed $k_{n}/k_{-}$ and $k_{+}k_{n}$ parameters in the Knowles’ aggregation model (Figure S1 in Supplementary Materials) [35]. The addition of pre-formed fibrils inhibited the average nucleation phase and enhanced the average elongation phase (Figure 1D), i.e., the secondary nucleation rate can be neglected. Further, increasing the hydrogelator decreased both secondary pathways rates, while GdL enhanced the monomer independent pathway and inhibited the monomer-dependent pathway (Table S2 in Supplementary Materials). Nonetheless, self-assembly can be majorly attributed to the primary pathways. Temperature exponentially affected the average nucleation and elongation phase, which is associated with a faster GdL proton dissociation (Figure 1E) [40]. As a result, overall gelation can be enhanced by increasing both GdL and hydrogelator concentrations and preparing the gel at 30 or 40 °C, which favors the primary pathways (nucleation and elongation).

The dye Nile Red was used to evaluate the effect of preparation conditions on the microenvironment, as it is a solvatochromic probe that has almost negligible emission in water, but intensely emits fluorescence in non-polar environments [41,42,43,44], as observed after hydrogel formation (Figure 1F). Furthermore, the emission is accompanied by a blue-shift with a reduction of polarity [41,42,43,44]. Here, Nile Red is localized in a microenvironment with a polarity between acetone and ethanol [41]. The higher Nile Red fluorescence emission intensity on the gels prepared at 30 °C and 40 °C suggests that more hydrophobic regions were made available. The fluorescence anisotropy values reveal similar fluidity compared to the hydrogel prepared at room temperature. Although increasing GdL (fixed hydrogelator concentration at 0.3 wt%) also contributed for more hydrophobic regions, the microfluidity was lower than that obtained by increasing the hydrogelator concentration (fixed GdL concentration at 0.5 wt%). As such, increasing both GdL and hydrogelator concentrations (0.4 hydrogelator-to-GdL ratio or higher) promotes more hydrophobic regions with higher microviscosity.

### 3.2. Nanoparticles Characterization

**Figure 2:**
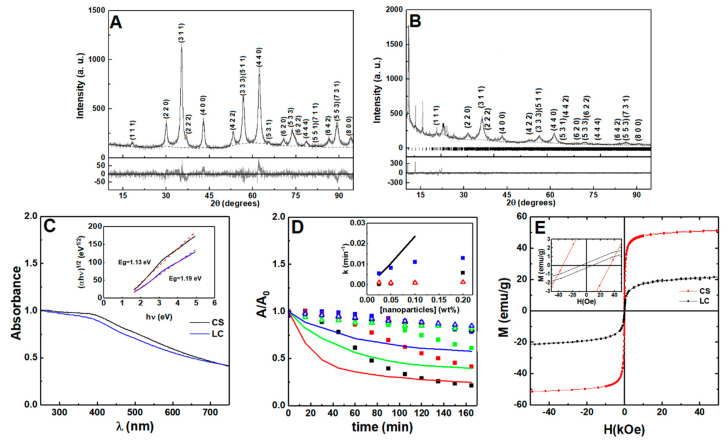
X-ray diffraction pattern of (**A**) citrate-stabilized and (**B**) lipid-coated manganese ferrite nanoparticles. Gray lines: experimental patterns; black lines: fitted patterns; dotted lines: fitted background; the vertical lines in (**B**) are the Bragg diffraction positions of manganese laurate. (**C**) Absorption spectra of citrate-stabilized (CS, black line) and lipid-coated (LC, blue line) MnFe_2_O_4_ nanoparticles. Inset: Tauc plot of citrate-stabilized and lipid-coated nanoparticles. (**D**) Sedimentation profiles of the citrate-stabilized (squares), lipid-coated (triangles), and bare (line) MnFe_2_O_4_ nanoparticles at 0.2 wt% (black), 0.1 wt% (red), 0.05 wt% (green), and 0.025 wt% (blue). Inset: sedimentation rate dependence on nanoparticle concentration. The citrate-stabilized aggregation rate is included (black squares). (**E**) Magnetization hysteresis loops of citrate-stabilized and lipid-coated manganese ferrite nanoparticles measured at room temperature (T = 300 K). Inset: Enlargement of the loops in the low field region.

Nanoparticles of manganese ferrite with different coatings were prepared using different synthetic methods, and are named, from now on, as citrate-stabilized or lipid-coated manganese ferrite nanoparticles. The X-ray diffraction (XRD) patterns of both samples present well-defined peaks (Figure 2A,B) characteristic of a crystalline structure, which was obtained without calcination. The diffraction peaks of the MnFe_2_O_4_ crystalline spinel structure are observed at 2θ = 29.7° (2 2 0), 34.9° (3 1 1), 36.5° (2 2 2), 42.5° (4 0 0), 52.7° (4 2 2), 56.2° (3 3 3) and (5 1 1), 61.8° (4 4 0), 65.0° (5 3 1), 70.1° (6 2 0), 73.1° (5 3 3), 74.0° (6 2 2), 78° (4 4 4), 85.6° (6 4 2), 88.5° (7 3 1) and (5 5 3), according to CIF file 2300618 (space group Fd-3m). Rietveld analysis was performed using the FullProf software suite, confirming the spinel structure. As in previous works [45,46], we considered it to be important to use microabsorption correction [47], resulting in fits with good R_F_ values. The calculated parameters are presented in Table S3 in Supplementary Materials, offering a larger size of the crystalline domains in the nanoparticles that are citrate-stabilized. Additional diffraction peaks were observed for the lipid-coated nanoparticles, occurring at positions similar to those reported for layered manganese laurate [48], which can be ascribed to ordered lauric acid molecules at the surface of manganese ferrite.

The UV-visible absorption spectra of the prepared nanoparticles are represented in Figure 2C. From the Tauc plot (inset of Figure 2C), the optical band gap (E_g_) between the citrate-stabilized and lipid-coated manganese ferrite nanoparticles was determined and a linear relation was obtained for an indirect semiconductor with a band gap of 1.13 eV and 1.19 eV, respectively, which are similar to the previous reported value of 1.08 eV [49].

The sedimentation profiles for bare, lipid-coated, and citrate-stabilized nanoparticles are displayed in Figure 2D. The dependence of the sedimentation rate on nanoparticle concentration (obtained through fitting of a Becquerel function or compressed hyperbola) [50] is included (values are reported in Table S4 in Supplementary Materials). The citrate-stabilized nanoparticles sedimentation profile suggests the occurrence of nanoparticles aggregation into stable agglomerates [51,52], which settle down at a faster rate than single nanoparticles. The lipid-coated nanoparticles show a sedimentation rate independent of the used concentration range (0.025–0.2 wt%). Hereby, the longer-term stability of the lipid-coated nanoparticles is expected to provide homogeneous gels at higher concentration of nanoparticles, compared to the ones that are citrate-stabilized.

The magnetic hysteresis loops show that the saturation magnetization (emu/g) is higher for the citrate-stabilized nanoparticles than for the lipid-coated nanoparticles (Figure 2E), which can be explained by taking a different stoichiometry into account in the manganese ferrite in the two samples, as pointed out by the different lattice constant obtained in the X-ray diffraction analysis. A smaller size and a higher wt% of organic matter present in the second sample can also influence the final value of saturation magnetization. Consequently, considering that SAR ∝ M_s_^2^ (SAR—specific absorption rate), the lipid-coated nanoparticles are expected to have lower heating efficiency than the citrate-stabilized nanoparticles [53,54]. The low M_r_/M_s_ ratio, of around 0.1 (see Table S5 in Supplementary Materials), is an indication that both types of nanoparticles display a superparamagnetic behavior [54].

### 3.3. Development of Magnetogels

**Figure 3:**
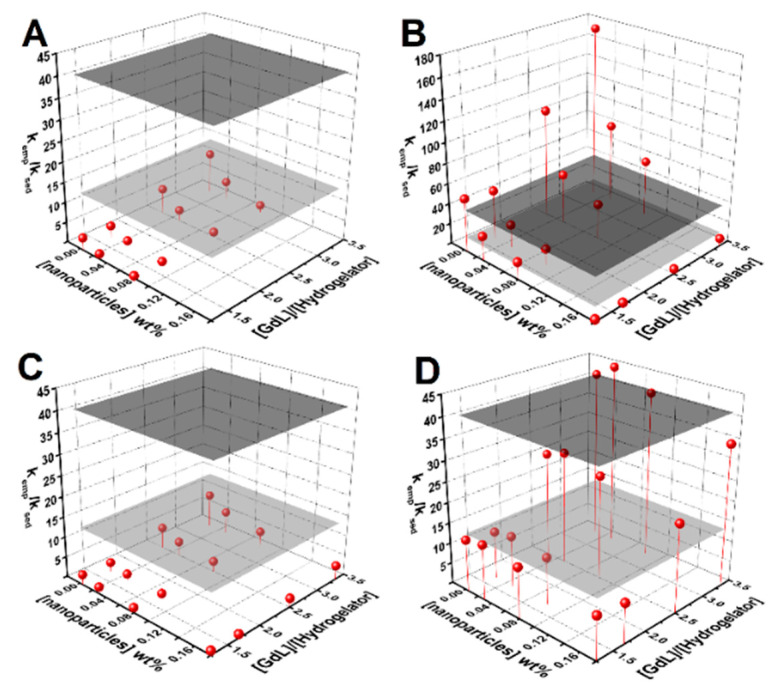
Surface plots of k_emp_/k_sed_ dependence on nanoparticle concentration and the GdL-to-hydrogelator concentration ratio for: (**A**) bare nanoparticles, (**B**) the citrate-stabilized nanoparticles aggregation rate, (**C**) the sedimentation rate, and (**D**) lipid-coated nanoparticles. The gray planes define the estimated k_emp_/k_sed_ required to guarantee that when the gel is about 10% of gelation completion, 90% of the nanoparticles remains in suspension (ν = 1, a = 0.5, dark gray plane; ν = 0.5, a = 0.5, gray plane).

An empirical equation was used to assist the estimation of the conditions required to maximize the homogeneity of the gel (see deduction, discussion and Figure S2 in Supplementary Materials). Figure 3 displays plots of the obtained k_emp_/k_sed_ for various nanoparticle concentration and GdL-to-hydrogelator concentration ratio. The estimation implies that the gelation conditions have to guarantee a k_emp_/k_sed_ > 41.7, so that when gel fraction *f(t)* attains 0.9, the nanoparticles suspended fraction is also at 0.9 (if v = 1 and a = 0.5 is assumed).

Gels prepared with 1.5 wt% GdL and 0.3 wt% hydrogelator retained the nanoparticles, though the pH value was lower than 4. Increasing the hydrogelator content to 0.5 wt% and reducing GdL to 1 wt% (has higher microviscosity) allowed the preparation of homogeneous magnetogels at 0.1 wt% of nanoparticles, with a pH of ~5. The 0.2 wt% content of nanoparticles can also be prepared at 0.5 wt% of hydrogelator and 1 wt% of GdL but preparation at 30 °C or 40 °C is required (yields k_emp_/k_sed_ larger than 200). Nonetheless, the parameter v obtained from curve fitting was around 0.5, thus decreasing the required k_emp_/k_sed_ to 14, which allowed the possibility of obtaining quasi-homogeneous gels at 0.1 wt% of nanoparticles at a [GdL]/[hydrogelator] ratio of 2 (see magnetogels and respective pH values in Figure S3 in Supplementary Materials).

### 3.4. Gels Microviscosity

**Figure 4:**
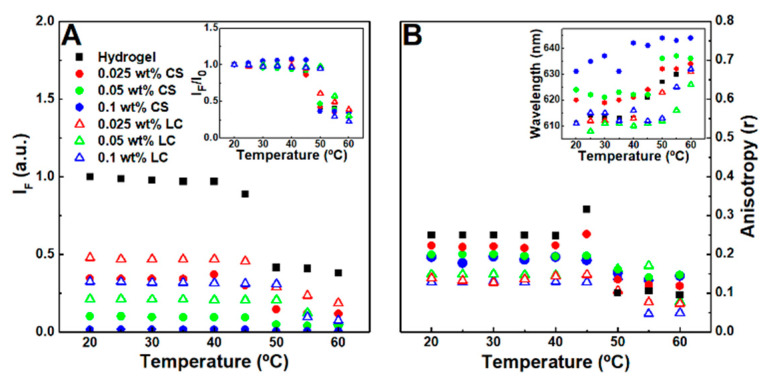
(**A**) Nile Red (2 µM) maximum fluorescence emission dependence on the temperature of hydrogel, citrate-stabilized (CS), and lipid-coated (LC) nanoparticles containing magnetogels. Inset: Normalized maximum fluorescence emission dependence on the temperature of the respective gels. (**B**) Nile Red fluorescence anisotropy dependence on temperature. Inset: Nile Red maximum emission wavelength dependence on temperature.

The effect of the nanoparticles in the gels matrix microenvironment was studied using Nile Red as a fluorescence probe, while considering its sensitivity to polarity and viscosity [55,56]. In the 20–40 °C temperature range, no major fluorescence emission decay changes were observed, which might be associated with the structure maintaining its integrity. A steep fluorescence emission decrease indicated that the phase transition occurs above 45 °C for the hydrogel and the citrate-stabilized nanoparticle-containing magnetogels, while in the lipid-coated nanoparticle-containing magnetogels, it was shifted to 50 °C (Figure 4A). The fluorescence anisotropy increases at the phase transition temperature, which might be associated with a fluorescence emission lifetime decrease (Figure 4B) [57].

The results suggest that citrate-stabilized nanoparticles destabilized the hydrophobic domains, leading to a microviscosity reduction and polarity increase of the cavities where Nile Red is localized, which might occur through hydrogen bonding and ionic interaction between the nanoparticles and the fibers. The polarity in the hydrogel is similar to acetone, which was changed towards ethanol after addition of citrate-stabilized nanoparticles. Furthermore, after phase transition and at 0.1 wt% of citrate-stabilized nanoparticles, the Nile Red emission wavelength (640 nm) became close to the reported maximum wavelength in water (657 nm) [42]. The lipid-coated nanoparticles induced a lower fluorescence anisotropy than citrate-stabilized nanoparticles and one that is similar to the reported anisotropy values of Nile Red in mixed vesicles and micelle membranes [44], thus suggesting that lipid-fiber domains are formed with a polarity similar to the fibers and a viscosity near that of membranes.

### 3.5. Gels Secondary Structure

**Figure 5:**
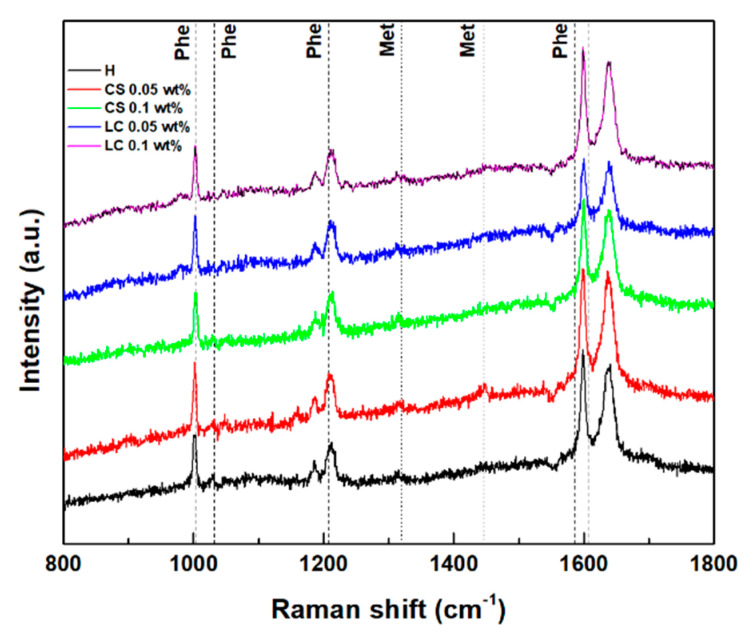
Raman scattering spectra of the hydrogel and magnetogels containing lipid-coated (LC) and citrate-stabilized (CS) nanoparticles. Vertical lines represent the reported Raman shifts of methionine (Met) and phenylalanine (Phe).

The Raman spectra of the hydrogels and magnetogels (0.1 wt% of nanoparticles) were obtained to assess influence of nanoparticles in the secondary structure, which are displayed in Figure 5. Reported Raman shifts of the phenylalanine phenyl ring and methionine side chain (CH_3_ deformation at 1440 cm^−1^ and CH_2_ wagging at 1320 cm^−1^) are also displayed [58,59]. Structural changes upon the addition of lipid-coated nanoparticles were suggested by the appearance of a band at 982 cm^−1^. Gaussian curves were fitted to the major phenyl ring signal at around 1003 cm^−1^ (see Figure S4 in Supplementary Materials). A blue shift and decreasing cross-section were observed with an increasing nanoparticle concentration. The latter effect can be associated with an increasing exposure of the aromatic rings to a more hydrated environment [60].

The Amide I region (1580–1700 cm^−1^) arises from the C=O stretching vibration, which is sensitive to changes in backbone peptide conformation [61]. Deconvolution of the amide I band in its sub-bands is correlated with various secondary structure contributions (see Figure S5 in Supplementary Materials) [60,61,62]. A major contribution at 1637 cm^−1^ is common to all systems, which can be associated with a major β-sheet content [61,62]. The band at 1338 cm^−1^ also confirms the predominance of the β-sheet in all gels [61].

### 3.6. Rheological Properties

Large amplitude oscillatory shear strain sweeps (LAOSS) were carried out to assess the effect of the nanoparticles on the gels structure (Figure S6 in Supplementary Materials). Further, the effect of temperature at 37 °C on the hydrogel shear elastic and loss moduli was also assessed (Figure S6), the decrease of which evidences the phase transition behavior observed in the microviscosity studies. The addition of nanoparticles reduced the elasticity of gels, as previously observed for other systems [10,63]. Interestingly, the lipid-coated nanoparticles induced a lower elasticity than the citrate-stabilized nanoparticles, which was similar to their influence on the gels’ microviscosity. Further, the strain at which G′ crosses G″ increased in the presence of citrate-stabilized nanoparticles (at 0.05 wt%). As a result, the profiles suggest that different structures were obtained for each nanoparticle content, compared to the bare gel. The lipid-coated nanoparticle-containing magnetogel displays the Payne effect characteristic of viscoelastic matrices reinforced by solid fillers, i.e., a local maximum of G″ concomitant with a significant decrease of G′, which can be associated with the breakage and recovery of weak interaction bonds linking adjacent clusters, aggregation/disaggregation of nanoparticles, or molecular disentanglement [64].

### 3.7. Electron Microscopy

**Figure 6:**
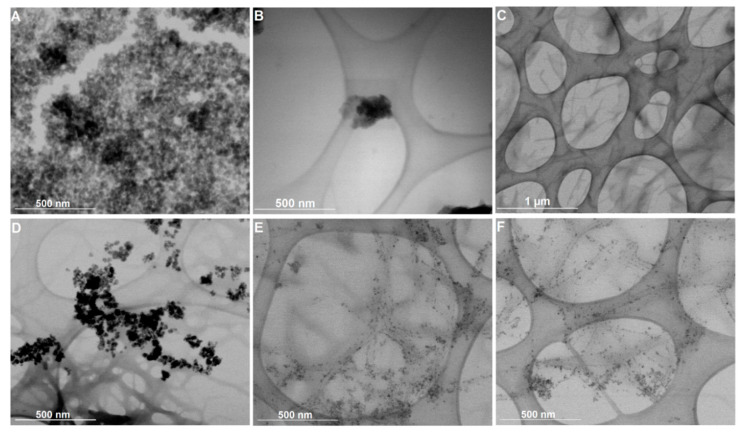
Scanning transmission electron microscopy (STEM) images of (**A**) citrate-stabilized nanoparticles, (**B**) lipid-coated nanoparticles, and (**C**) the hydrogel structure. Magnetogels containing (**D**) citrate-stabilized nanoparticles and (**E**,**F**) lipid-coated nanoparticles prepared at 0.5 wt% of hydrogelator, 1 wt% of GdL, and 0.05 wt% of nanoparticles.

Figure 6 displays the STEM images of the citrate-stabilized and lipid-coated nanoparticles in solution and incorporated into the hydrogel matrix (magnetogels) prepared at 0.5 wt% of hydrogelator. The negative charge of the citrate molecules stabilizing the nanoparticles ensured there were well dispersed nanoparticles with an average size of 14.4 ± 2.6 nm (see histograms in Figure S7 in Supplementary Materials), though some aggregates were observed (Figure 6A). Alternatively, the lipid-coated nanoparticles, with an average size of 8.9 ± 2.1 nm, displayed a tendency to form spherical aggregates, which can be associated with the dynamic membrane coating being prone to self-assembly (Figure 6B). The hydrogel network is also displayed for a matter of comparison with the magnetogels. The preparation conditions used here afforded a network comprised of thin and thick short fibers with a cross-section of 21.3 ± 3.4 nm and 48.4 ± 13.8 nm, and with an average length of 1030 ± 389 nm.

Furthermore, different effects on the magnetogels microstructure were observed when using the two types of nanoparticles. The citrate-stabilized nanoparticles are randomly distributed within the hydrogel matrix as aggregates. This stems from the fact that the magnetogels based on N-protected peptides lacked a cationic group in the hydrogelator structure electrostatically interacting with these negatively-charged stabilized nanoparticles and fixing them in the matrix. Nevertheless, the lipid-coated nanoparticles displayed an affinity towards the fibers surface, rendering them more adequate to avoid any potential leaking of nanoparticles. Furthermore, upon gelation, the lipid-coated nanoparticle aggregates become destabilized, as no aggregates were observed (see Figure 6E,F).

### 3.8. Hyperthermia Studies

**Figure 7:**
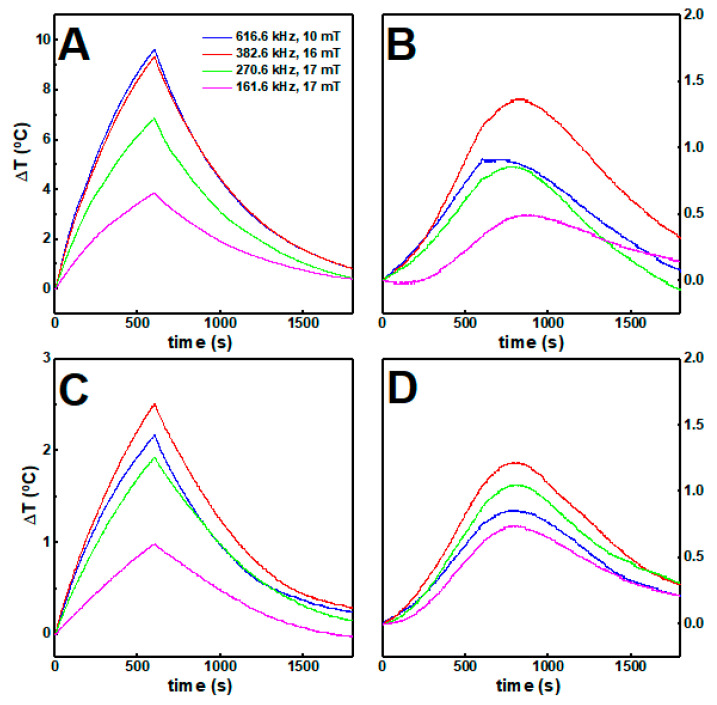
The temperature variation over time of (**A**) citrate-stabilized and (**B**) lipid-coated nanoparticles at 1 mg/mL (0.1 wt%) in water and in gels (**C**,**D**) respectively) under different magnetic field strengths and frequencies.

The calorimetric approach was carried out to assess the magnetic nanoparticle hyperthermia effect in gels, while considering the medical threshold limit of $H_{0}f$ ≤ 5 × 10^9^ A m^−1^ s^−1^ [65,66], or $H_{0}f$ ≤ 4.85 × 10^8^ A m^−1^ s^−1^ [66], depending on the area exposed. The increase in temperature over time, when the nanoparticles are dispersed in aqueous solution and in gels while applying an alternating magnetic field, is displayed in Figure 7.

In water solution, the citrate-stabilized nanoparticles attained higher temperatures than the lipid-coated nanoparticles, which could be associated with the different stoichiometry in the spinel ferrites belonging to the two samples, with the different average sizes, and with the fact that the lipid-coated nanoparticles are more likely to aggregate in aqueous solution. In both cases, the increments in temperature decreased when the magnetic nanoparticles were incorporated in the gels, likely because some of the heat generated was employed for inducing local changes in the gel structure.

Furthermore, nanoparticles at high concentration within the gels underwent stronger magnetic dipolar interactions, which has a detrimental effect in the heat delivery capacity [67,68]. The heating efficiency was quantitatively evaluated through the intrinsic loss power (ILP) (see Table S6 in Supplementary Materials). While the ILP decreases for the citrate-stabilized nanoparticles around 80% when incorporated into the gels, for the lipid-coated nanoparticles no major changes were obtained. Accordingly, despite being less efficient than citrate-stabilized nanoparticles, the lipid-coated nanoparticles keep a similar heat delivery capacity when they are incorporated in the gels.

### 3.9. Drug Release Assays

#### 3.9.1. Incorporation of Doxorubicin

Previously, supramolecular dehydropeptide-based hydrogels have shown promising results as drug delivery nanosystems [8]. Here, the nanoparticles concentration effect over doxorubicin incorporation is assessed. FRET (Förster Resonance Energy Transfer) process from the emissive moieties of the hydrogel aromatic moieties (acting as the energy donors) to doxorubicin (acting as the energy acceptor) allowed us to follow the encapsulation of doxorubicin owing to the overlap between the drug absorption band and hydrogel fluorescence emission (see Figure S8 in Supplementary Materials).

**Figure 8:**
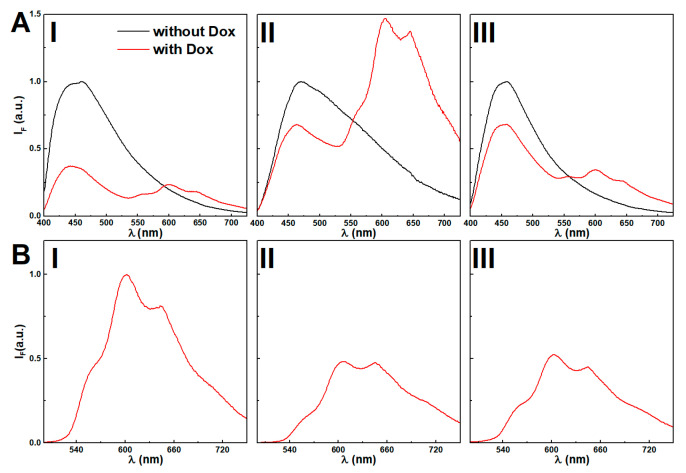
(**A**) Fluorescence emission spectra (λ_exc_ = 375 nm) of hydrogel (I) and magnetogels containing citrate-stabilized (II) and lipid-coated (III) manganese ferrite nanoparticles (0.025 wt%) incorporating the doxorubicin and comparison with the plain gels. (**B**) Fluorescence spectra (λ_exc_ = 480 nm) of directly-excited doxorubicin in gels, (I) and magnetogels con-taining citrate-stabilized (II) and lipid-coated (III) manganese ferrite nanoparticles (0.025 wt%) incorporating the doxorubicin and comparison with the plain gels

Fluorescence spectra of hydrogel and magnetogels at 0.025 wt% of citrate-stabilized and lipid-coated nanoparticles, with and without doxorubicin, are displayed in Figure 8. The absence of doxorubicin results in a strong fluorescence emission (Figure 8A) of the aggregates associated with the stacking of the aromatic rings (λ_max_ ~ 450 nm).

The addition of citrate-stabilized nanoparticles induced a red-shift (Figure 8B), which was modulated by increasing the nanoparticle concentration (see Figure S9 in Supplementary Materials). The lipid-coated nanoparticles induced a thinning of the emission band shape, suggesting that the variety of microenvironments in the vicinity of the fibers was restrained.

Doxorubicin displays reverse solvatochromism, making the assignment of its location ambiguous [69]. Further, the assays with Nile Red demonstrated that a higher concentration of citrate-stabilized nanoparticles was observed to increase the polarity of the gel hydrophobic cavities. Thus, the decreasing fluorescence emission ratio of doxorubicin between the peaks at λ = 560 nm and λ = 600 nm upon addition of citrate-stabilized nanoparticles might be associated with an increasingly acidic region and its location in cavities, which are destabilized by the increasing concentration of nanoparticles (see Figure S10 in Supplementary Materials) [69]. Nonetheless, the possibility of aggregation is not excluded, considering that at 10 µM, a fraction of 47% is dimerized [70,71], and the obtained spectra in gels resemble the fluorescence emission spectrum of a doxorubicin concentrated solution (0.1 mM) at pH = 7 (see Figure S11 in Supplementary Materials). The lipid-coated nanoparticles do not show the same effects, as the wavelength and emission ratio remain mostly unchanged, i.e., no major changes are induced in the doxorubicin location microenvironment. The fluorescence quenching by increasing nanoparticle concentration further suggests its proximity to the nanoparticles.

The hydrogelator fluorescence quantum yield at a nanoparticle concentration of 0.025 wt% was determined through Equation (7). The calculated FRET efficiencies ($\Phi_{\mathrm{FRET}}$), Förster radius ($R_{0}$), and donor-acceptor distances ($R_{DA}$) are presented in Table 1. As reported in other systems [8,63], the distances between the fiber aromatic groups and doxorubicin remain similar with or without nanoparticles, and suggests a host-guest type interaction [8,63].

Here, similarly to the obtained results with Nile Red, the lipid-coated nanoparticles induced a lower anisotropy, while the fluorescence emission spectrum remains similar, suggesting the occurrence of lipid-fiber domains. Overall, the high anisotropy values suggest that doxorubicin has affinity towards the gel network fibers and the nanoparticles affect the arrangement of the fibers.

#### 3.9.2. Doxorubicin Release Assays

In a previous drug release assay of hydrogels, a mixed behavior of gel erosion and drug diffusion was observed [8]. Doxorubicin release from the hydrogel and magnetogels to pH = 7 buffer (to keep pH conditions constant and neutralize gels) was assessed.

**Figure 9:**
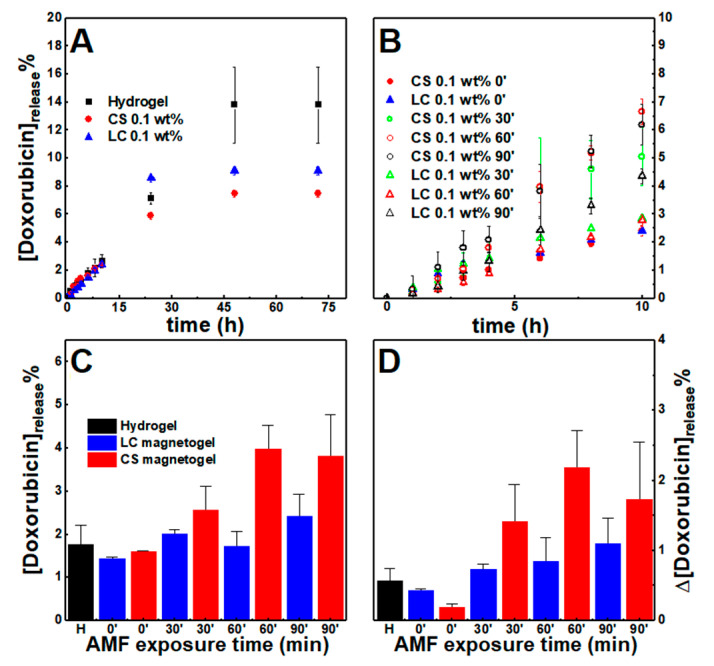
Cumulative doxorubicin percentage release profiles from the hydrogel and gels containing citrate-stabilized (CS) and lipid-coated (LC) nanoparticles (0.1 wt%) (**A**) without an externally applied alternating magnetic field (AMF) and (**B**) comparison of the magnetogels release when an AMF is applied for 30, 60, and 90 min between t = 4 h and t = 6 h. (**C**) Cumulative doxorubicin released at t = 6 h. (**D**) Variation of doxorubicin percentage released between t=4 h and t = 6 h, that is, for a period of 2 h.

The release profiles of the hydrogel and both limiting conditions (0.1 wt% of nanoparticles) of magnetogels are displayed in Figure 9A. Similarly to previous results for dehydropeptide hydrogels (without nanoparticles) and other magnetogels [62], an initial burst release occurs, followed by a slow release phase. Moreover, both systems display a low drug release profile that can be associated with the strong interactions established between the drug and gels components. Such profiles are useful for therapeutic applications, as the systems can be loaded with high amounts of chemotherapeutic drugs and ensure a prolonged and controlled release of a therapeutically relevant dose in the target site.

Quantitative analysis of the cumulative drug release profiles without a magnetic field was carried out through the fitting of various mathematical models (see Tables S7 and S8 in Supplementary Materials) [72,73,74,75].

Overall, the obtained Gompertz model fitting suggests that both nanoparticles limited the maximum drug quantity released, while the Korsmeyer-Peppas mechanistic model (0.45 < n < 0.89) points to a mechanism that combines diffusion and erosion drug release (non-Fickian release) [72,73,74]. The latter contribution was also evidenced by the good fitting of the first-order model (associated with the diffusion of water-soluble drugs in porous matrices) and the Hixson-Crowell model (describes systems where changes in the surface area and diameter occur over time, but the initial geometrical shape is kept constant).

An alternating magnetic field (AMF) was applied for 30, 60, and 90 min, 2.98 mT at 1000 kHz after 4 h, which resulted into an increase of cumulative doxorubicin release comparatively to the systems that were not subjected to the AMF (Figure 9B,C). To remove the contribution from preparation anomalies, the enhancement of drug release was further evidenced by comparing the amount of drug release in the period between t = 4 h and t = 6 h (Figure 9D), that is, during two hours. The enhancement is larger in the citrate-stabilized nanoparticles than lipid-coated nanoparticles, which might be associated with the higher heating efficiency. However, the enhanced drug release displays a non-linear relation with the AMF exposure time. This can be associated with the nanoparticles being distributed as aggregates, as opposed to the lipid-coated nanoparticles that incrementally enhanced drug release, as the sample was exposed to longer periods. Further, gels subjected to the AMF for 30 min revealed a decrease of fluorescence anisotropy to 0.12 for the citrate-stabilized nanoparticles, while in the lipid-coated ones, it remained close to 0.15, i.e., the citrate-stabilized nanoparticles heating induced an irreversible collapse of the gel network that led to an increased variance of drug released, while the other nanoparticles might have not affected the network structure.

As a result, the incremental drug release enhancement and retention of the lipid-coated nanoparticles developed here indicate that this supramolecular system architecture is a suitable approach for controlling drug release, since the heating-induced effect displayed improved reproducibility. However, lipid-coated nanoparticles with a higher heating efficiency and gels that can homogeneously include more nanoparticles are required in future developments to improve the percentage of drug released upon the AMF-trigger, as suggested from the higher triggered release from citrate-stabilized nanoparticle-containing gels.

## 4. Conclusions

Pursuing the effect of citrate and lipid-functionalized nanoparticles in the development of supramolecular magnetogels, the gelation of the hydrogel Cbz-L-Met-*Z*-ΔPhe-OH was systematically optimized by using kinetic models to prepare homogeneous magnetogels, while considering both the kinetics of gelation and sedimentation of nanoparticles. Lipid-coated nanoparticles formed lipid-fiber domains and increased the gel irreversible phase transition temperature. The heating efficiency of lipid-coated nanoparticles was improved (maximum heat and reproducibility) when they were incorporated in the gels, while a detrimental effect was obtained for citrate-stabilized nanoparticles. Further, the former did not produce major changes in doxorubicin encapsulation, while the latter increased the micropolarity of its location and induced aggregation. The magnetogels revealed similar doxorubicin release profiles and AMF-trigger was stronger in the citrate-stabilized nanoparticles, though the triggered release was more reproducible in the lipid-coated nanoparticle-containing gels.

Overall, the lipid-coated nanoparticles displayed promising results for future developments of supramolecular magnetogels aiming at the control of drug release. This was mainly associated with the improved nanoparticle distribution (along the hydrogel fibers), unaffected heating efficiency upon gelation, and reproducible triggered drug release. On the other hand, despite the higher heating efficiency of the negatively-charged citrate-stabilized nanoparticles in solution, they are prone to aggregation upon gelation, which is reflected in a decreased heating efficiency, and local inhomogeneous distribution consequently leading to less reproducibility in drug release.

Hereby, this work reveals that negatively-charged stabilized and lipid-coated nanoparticles affect the final gel architecture differently and, thus, also affect its properties and the encapsulation of drugs in different ways. Further, comparison of both systems points out that on-demand drug release in dehydropeptide-based supramolecular magnetogels can be optimized by developing nanoparticles that can adsorb onto hydrogel fibers, while providing domains that improve or do not affect drug encapsulation (lipid-coated nanoparticles). In particular, the unaffected drug encapsulation and reproducible release from the lipid-coated nanoparticle-containing gels, upon application of AMF, is anticipated to potentiate the supramolecular magnetic gels in drug delivery towards on-demand drug release.

Future developments will be focused on improving heating efficiency of the lipid-coated nanoparticles and the synergy between hyperthermia and triggered drug release, without inducing a major collapse of the hydrogel.

## Figures and Tables

**Table 1:** Förster Resonance Energy Transfer (FRET) efficiencies ($\Phi_{FRET}$), fluorescence quantum yields of the donor (hydrogel aggregates) ($\Phi_{D}^{0}$), donor-acceptor distances ($R_{DA}$) and steady-state fluorescence anisotropy (r) values for gels with an incorporated drug. Anisotropy value of doxorubicin in glycerol (at 25 °C): r = 0.285.

System	Content (wt%)	$\Phi_{FRET}$	$\Phi_{D}^{0}{\;}^{a}$	$R_{0}\;(\mathrm{nm})$	$R_{DA}\;(\mathrm{nm})$	r
H	-	0.66	0.012	2.1	1.9	0.17
CS	0.025	0.33	0.001	1.7	1.4	0.16
	0.05	-	-	-	-	0.15
	0.1	-	-	-	-	0.14
LC	0.025	0.33	0.010	2.3	2.0	0.13
	0.05	-	-	-	-	0.11
	0.1	-	-	-	-	0.11

^a^ Relative to L-tryptophan in aqueous buffer solution, pH = 7.2 ($\Phi_{r}=0.14$ at 25 °C) [39]. The error rate is about 10%.

## Supplementary Materials

The following are available online at https://www.mdpi.com/2079-4991/11/1/16/s1, Structure S1: Hydrogelator structure, Table S1: Final hydrogel pH, Figure S1: Knowles’ aggregation model fitting to experimental aggregation profiles, Table S2: Calculated curve-fitting parameters of the turbidity profiles, Table S3: Calculated X-ray diffraction parameters, Table S4: Calculated curve-fitting parameters of the sedimentation profiles, Table S5: Obtained parameters from the SQUID hysteresis curves, Figure S2: dependence of $\frac{k_{emp}}{k_{sed}}$ on s(t) and f(t) for fixed v and a values, Figure S3: Image of the magnetogels containing citrate-stabilized and lipid-coated nanoparticles, Figure S4: Curve-fitting of the Raman scattering spectra in the wavelength range 960–1040 cm^−1^ and dependence of the Raman shift and full width at half maximum of the major phenyl ring vibration, Figure S5: Curve-fitting of the Raman scattering spectra of gels in the amide I region, Figure S6: Strain dependence of the shear elastic and loss moduli for gels, Figure S7: STEM images and histograms of gels, Table S6: Calculated magnetic hyperthermia parameters, Figure S8: Normalized fluorescence emission spectrum of the hydrogel and doxorubicin absorption spectrum, Figure S9: Normalized fluorescence emission spectra of hydrogel and magnetogels, Figure S10: Fluorescence emission spectra of doxorubicin in hydrogel and magnetogels at various nanoparticle concentration, Figure S11: Normalized fluorescence emission spectra of doxorubicin in pH = 7 buffer at various concentrations, Table S7: Coefficients of determination for doxorubicin release profiles, Table S8: Release coefficients of the models fitted to the experimental release profiles.
