# Supplementary material for: Impact of Citrate and Lipid-Functionalized Magnetic Nanoparticles in Dehydropeptide Supramolecular Magnetogels: Properties, Design and Drug Release
Source: Nanomaterials (Basel). 2020 Dec 23;11(1):16. doi: 10.3390/nano11010016 (PMC7824179; doi:10.3390/nano11010016)
Supplement: Supplementary file 1 [file nanomaterials-11-00016-s001.pdf]

Impact of Citrate and Lipid-Functionalized Magnetic Nanoparticles in Dehydropeptide Supramolecular Magnetogels: Properties, Design and Drug Release

Sérgio R. S. Veloso [1], Joana F. G. Silva [1], Loic Hilliou [2], Cacilda Moura [1], Paulo J. G. Coutinho [1], José A. Martins [3], Martín Testa-Anta [4,5], Verónica Salgueiriño [4,5], Miguel A. Correa-Duarte [5], Paula M. T. Ferreira [3] and Elisabete M. S. Castanheira [1,*]

[1] Centro de Física (CFUM), University of Minho, Campus de Gualtar, 4710-057 Braga, Portugal; sergioveloso96@gmail.com (S.R.S.V.); joanasilva.fg@gmail.com (J.F.G.S.); cmoura@fisica.uminho.pt (C.M.); pcoutinho@fisica.uminho.pt (P.J.G.C.)

[2] Institute for Polymers and Composites, Department of Polymer Engineering, University of Minho, Campus de Azurém, 4800-058 Guimarães, Portugal; loic@dep.uminho.pt

[3] Centro de Química (CQUM), University of Minho, Campus de Gualtar, 4710-057 Braga, Portugal; jmartins@quimica.uminho.pt (J.A.M.); pmf@quimica.uminho.pt (P.M.T.F.)

[4] Departamento de Física Aplicada, Universidade de Vigo, 36310 Vigo, Spain; mtesta@uvigo.es (M.T.-A.); vsalgue@uvigo.es (V.S.)

[5] CINBIO, Universidade de Vigo, 36310 Vigo, Spain; macorrea@uvigo.es

* Correspondence: ecoutinho@fisica.uminho.pt; Tel.: +351-253-604321

Supplementary materials

Hydrogelator structure

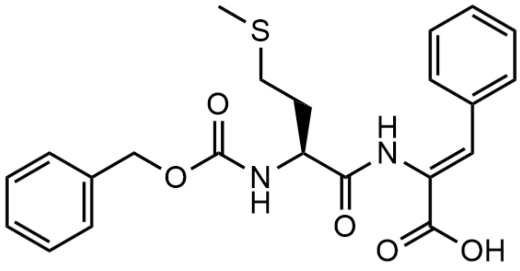

Structure S1. Cbz-L-Met-Z-ΔPhe-OH hydrogelator chemical structure. Legend: Cbz: carboxybenzyl; Met: methionine; ΔPhe: dehydrophenylalanine.

Final hydrogel pH

Table S1. Final hydrogel pH values obtained for variable GdL and hydrogelator concentrations (initially dissolved in 2 v/v% NaOH 1M).

Hydrogelator (wt%)	GdL (wt%)	pH
0.3	0.5	5.1
0.3	1	3.6
0.3	1.5	3.4
0.5	0.8	5.5
0.5	1	5.4
0.8	0.5	5.3

Hydrogel concentration gelation dependence

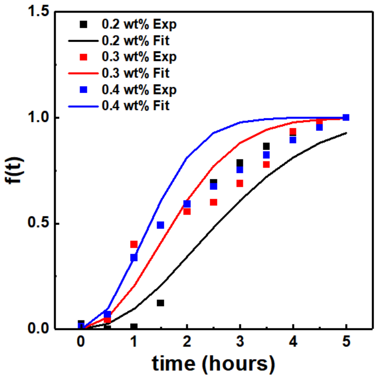

Figure S1. Knowles' aggregation model fitting (Fit) to experimental (Exp) aggregation profiles at fixed $\frac{k_n}{k_-} = 13706.3\ M^{-1}\ s^{-2}$, $k_+k_n = 0.35\ \mathrm{M}^{-1}\ \mathrm{s}^{-2}$ and $n_c = 2$ parameters. $R^2\ (0.2\ wt\%) = 0.86$, $R^2\ (0.3\ wt\%) = 0.92$ and $R^2\ (0.2\ wt\%) = 0.94$.

Table S2. Curve-fitting parameters obtained from the fitting of the aggregation models to the turbidity profiles. Legend: H: Hydrogel wt%; GdL: Glucono-δ-lactone wt%; F: Pre-formed fibrils wt%; T: Temperature.

Model	Empirical	Sâito's			Knowles'		Cohen's	
H/GdL/F/T	$k_{emp}\ (h^{-1})$	k_n (h^{-1})	k_p $(M^{-1}h^{-1})$	k_s (h^{-1})	k_n/k_- $(M^{-1}\ h^{-2})$	k_-k_+ $(\mathrm{M}^{-1}\ h^{-2})$	k_+k_n $(M^{-2}\ h^{-2})$	$k_2k_+ (M^{-3}\ h^{-2})$
0.2/0.5/0/20	1.73	0.03	404.67	1.89	111.53	50.88	8823.32	11682557.76
0.3/0.5/0/20	0.78	0.28	65.33	0.46	5174.13	0.70	2775.79	3245.75
0.4/0.5/0/20	0.85	0.28	56.72	0.53	4011.94	0.58	1796.56	2346.11
0.3/1/0/20	2.77	0.49	161.60	1.51	2316.08	8.25	14673.14	24981.28

0.3/1.5/0/20	254.27	1.38	215.14	2.01	280.75	286.15	50598.43	3091.88
0.3/0.5/5/20	1.84	0.08	175.83	1.64	913.26	4.57	5534.91	3350761.76
0.3/0.5/10/20	1.42	0.03	152.99	1.43	39.52	35.83	2168.12	2356519.30
0.3/0.5/0/30	1.25	0.22	83.51	0.78	4128.42	1.05	5987.92	1345960.84
0.3/0.5/0/40	321.28	0.90	504.28	4.71	20062.77	5.11	137506	12822707.97

X-ray diffraction parameters

A Rietveld analysis was performed using a secondary phase adapted from a CIF file of lead laurate (CIF file 7228429) by substituting lead by manganese. A reasonable result was obtained considering that the actual nanoparticle structure does not correspond to the independent existence of metal ferrite and layered metal laurate crystal structures, but rather to an ordered layer of laurate molecules connected to metal ions at the surface of a spinel crystal.

Table S3. X-ray diffraction Rietveld refinement calculated parameters R_f, χ^2 and phase sizes.

**Nanoparticles**	**Micro absorption correction (#)**		**Phase size (nm) ǀ ǀ Lattice Constant (Å)**	**R_f**	**χ^2**
Citrate-stabilized	P_0 = 0.72	τ = 0.084	8.3 ǀ 8.428	2.91	0.963
Lipid-coated	P_0 = 0.42	τ = 0.179	4.3 ǀ 8.410	5.28	2.11
				38.8 (*)	

(#) equation (8) in [47] with C=1 (*) Mn laurate phase.

Nanoparticle sedimentation

Table S4. Curve-fitting parameters obtained from the fitting of the Becquerel decay function to the sedimentation profiles. Legend: k_{agg}: aggregation rate; k_{sed}: sedimentation rate.

**Nanoparticle**	**Concentration (wt%)**	**k_{agg} (min^{-1})**	**k_{sed} (min^{-1})**
Bare	0.1	-	0.0237
	0.05	-	0.0104
	0.025	-	0.0045
Lipid-coated	0.2	-	0.0013
	0.1	-	0.0011
	0.05	-	0.0010
	0.025	-	0.0011
Citrate-stabilized	0.2	0.0058	0.0130
	0.1	0.0008	0.0111
	0.05	0.0006	0.0082
	0.025	0.0003	0.0055

SQUID results

Table S5. Coercive field (H_c), saturation magnetization (M_s), remnant magnetization (M_r), and ratio M_r/M_s for citrate-stabilized (CS) and lipid-coated (LC) nanoparticles, at room temperature (T=300 K).

	H_c (Oe)	M_s (emu/g)	M_r (emu/g)	M_r/M_s
**CS**	33.4	52.0	5.4	0.104
**LC**	8.0	21.4	0.2	0.009

Estimation of magnetogel gelation conditions

The gelation profile can be described according to the following general sigmoidal equation (2):

$$T(t) = \frac{T(\infty)}{\sqrt[v]{(1 + ve^{-k_{emp}(t-t_m)})}}$$

where $T(t)$ is the turbidity at time t, $T(\infty)$ is the final turbidity, k_{emp} is the rate constant (inverse of the relaxation time) of fibril formation and t_m is the point of the maximum elongation rate. Equation 4 can be re-written as:

$$ln\left(\frac{\frac{1}{f(t)^{\frac{1}{v}}}-1}{v}\right) = -k_{emp}(t - t_m) \qquad \text{(S1)}$$

$$f(t) = \frac{T(t)}{T(\infty)} \qquad \text{(S2)}$$

The sedimentation profiles can be initially described by a first-order sedimentation rate:

$$s(t) = e^{-k_{sed}t} \qquad \text{(S3)}$$

$$-\frac{ln(s(t))}{k_{sed}} = t \qquad \text{(S4)}$$

where $s(t)$ is the normalized absorption, $\frac{A(t)}{A(0)}$, measured at time t and k_{sed} is the sedimentation rate constant. By substitution of equation S4 into equation S1 and assuming t_m occurs at a fraction a of t the following equation is obtained:

$$ln\left(\frac{\frac{1}{f(t)^{\frac{1}{v}}}-1}{v}\right)\frac{1}{ln(s(t))}\frac{1}{(1-a)} = \frac{k_{emp}}{k_{sed}} \qquad \text{(S5)}$$

Equation S5 allows the empirical estimation of highest sedimentation rate constant for a given empirical gelation profile to guarantee a homogeneous supramolecular magnetogel, and consequently the maximum concentration of nanoparticles if the k_{sed} dependence on nanoparticle concentration is known. It is also possible to estimate the required gelation kinetic rate required for a given sedimentation rate if some assumptions are made for the parameters v and a. In figure S2 is demonstrated the dependence of $\frac{k_{emp}}{k_{sed}}$ on $s(t)$ and $f(t)$ for fixed v and a values. Here, only the positive ratio values have physical meaning, where increasing a and v results into an increase of the ratio for higher $s(t)$ values and fixed $f(t)$, i.e. to attain at a certain time a gel $f(t)$ fraction with $s(t)$ suspended nanoparticles the gelation rate has to increase for higher parameters.

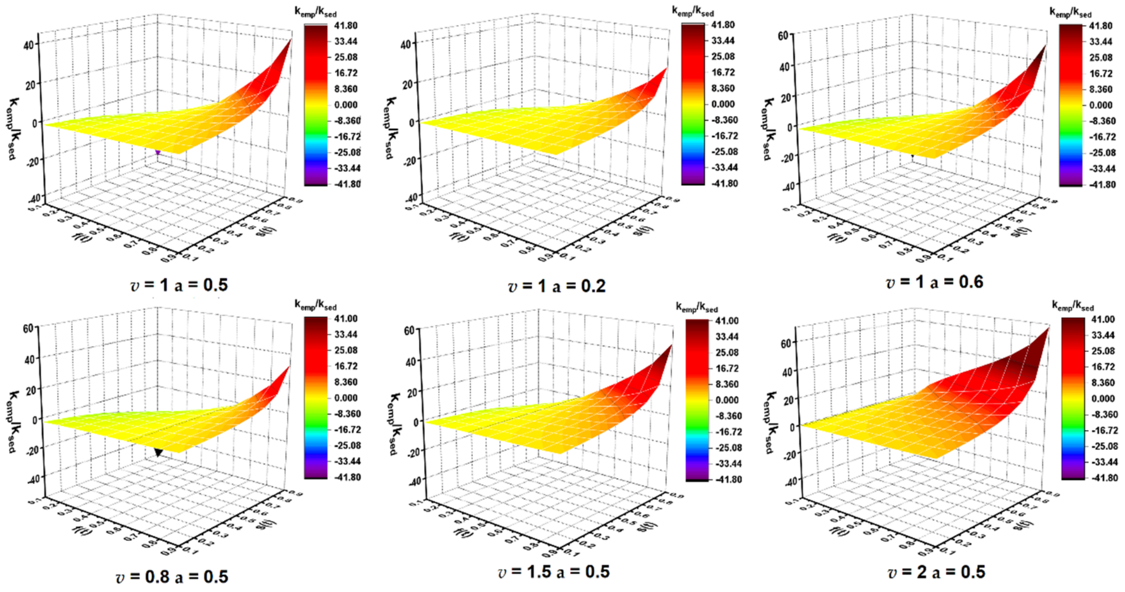

Figure S2. Dependence of $\frac{k_{emp}}{k_{sed}}$ on $s(t)$ and $f(t)$ for fixed v (0.8, 1, 1.5 and 2) and a (0.2, 0.5 and 0.6) values.

Hereby, if the required $\frac{k_{emp}}{k_{sed}}$ is estimated using the gelation profile parameters $v = 1$ and $a = 0.5$, where the gel fraction $f(t)$ has to be higher than 0.9 and the nanoparticles suspended fraction is 0.9, only the gelation conditions with $\frac{k_{emp}}{k_{sed}} > 41.7$ are expected to provide higher homogeneity, while for $v = 0.5$ the required ratio reduces to 14.3.

Limiting magnetogel gelation conditions

Magnetogels containing citrate-stabilized nanoparticles could be formed at 0.1 wt% of nanoparticles and 1 wt% of GdL at 0.5 wt% of hydrogelator. Increasing the nanoparticle content to 0.2 wt% resulted in the gelation inhibition, requiring higher GdL content to obtain gels. The lipid-coated nanoparticles did not show the same effect as gels could be obtained at 0.2 wt%.

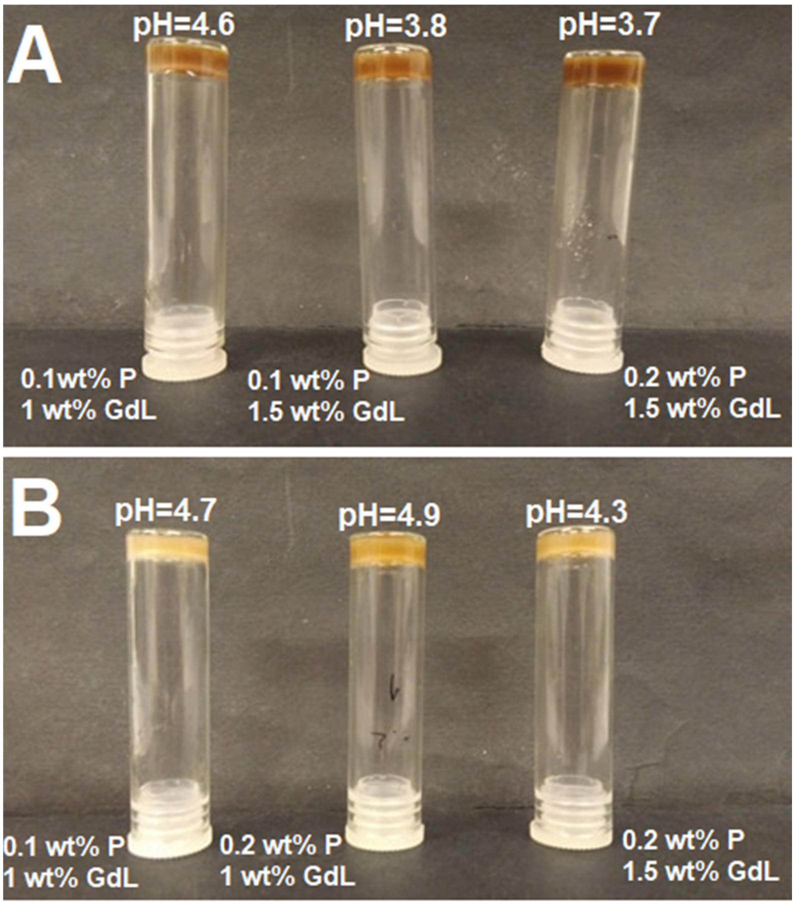

Figure S3. Image of the magnetogels containing citrate-stabilized (A) and lipid-coated (B) nanoparticles at 0.5 wt% of hydrogelator and the respective final pH.

Effect of nanoparticles on the fibers secondary structure

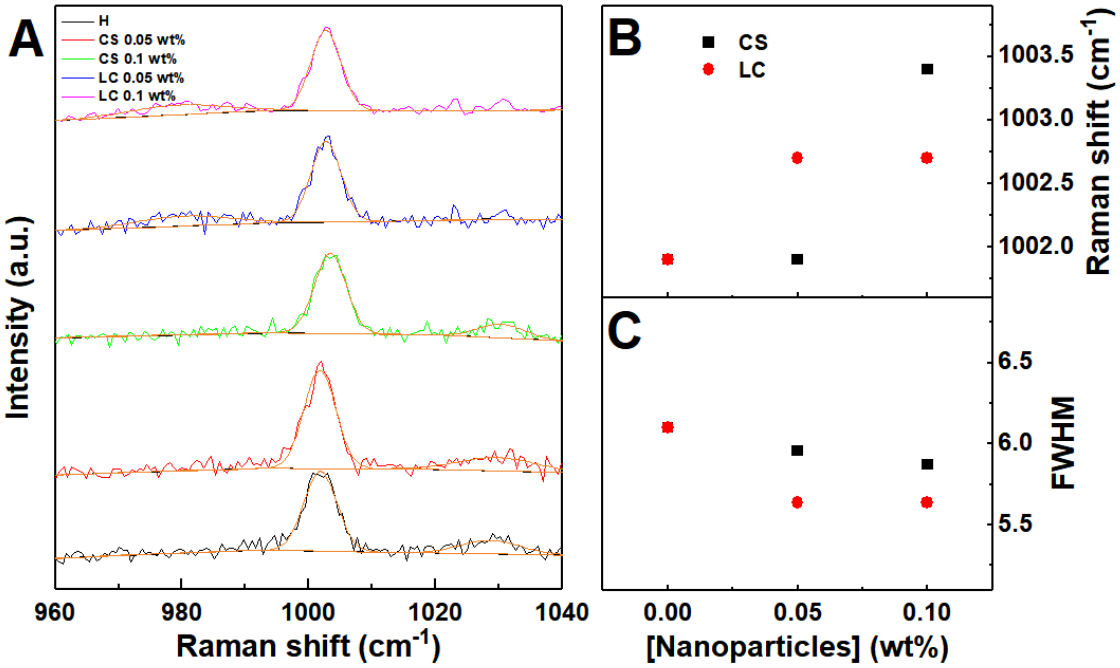

Figure S4. (**A**) Curve-fitting of the Raman scattering spectra of the hydrogel and magnetogels containing lipid-coated (LC) and citrate-stabilized (CS) nanoparticles in the wavelength range 960-1040 cm^{-1}. Dependence of: (**B**) Raman shift of the major phenyl ring vibration and (**C**) full width at half maximum (FWHM) of the fitted curve on the nanoparticles concentration.

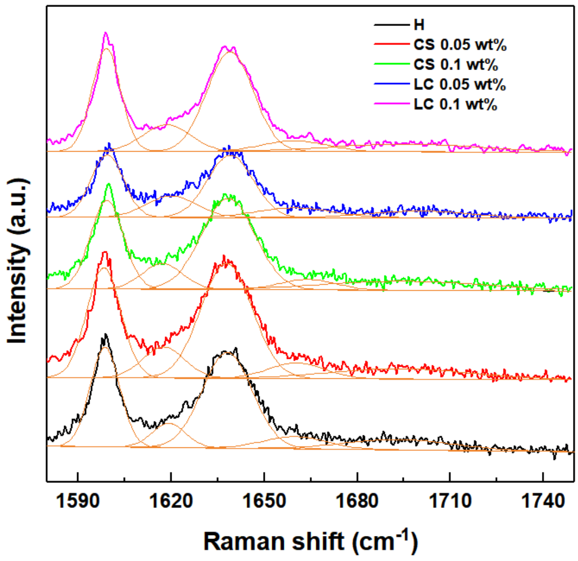

Figure S5. (**A**) Curve-fitting of the Raman scattering spectra of the hydrogel and magnetogels containing lipid-coated (LC) and citrate-stabilized (CS) nanoparticles in the amide I region.

Effect of nanoparticles on elasticity

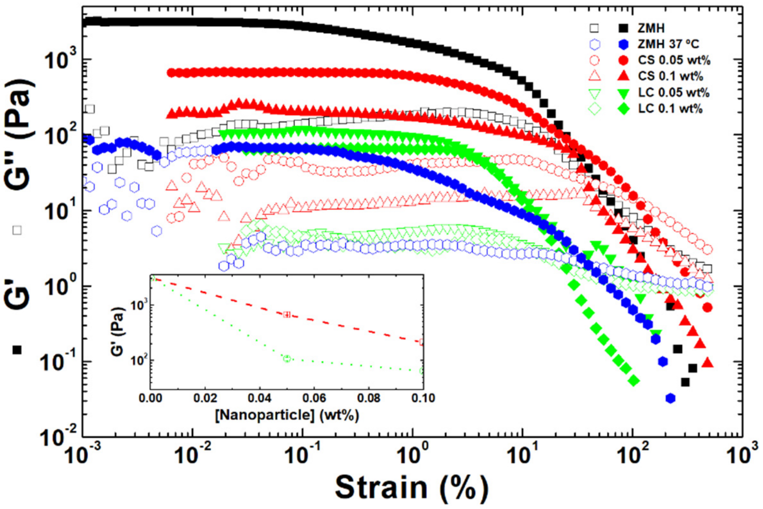

Figure S6. Strain dependence of the shear elastic (filled symbols) and loss moduli (empty symbols) for gels formulated at 0.5 wt%, 1 wt% of GdL and variable nanoparticle content.

STEM histograms

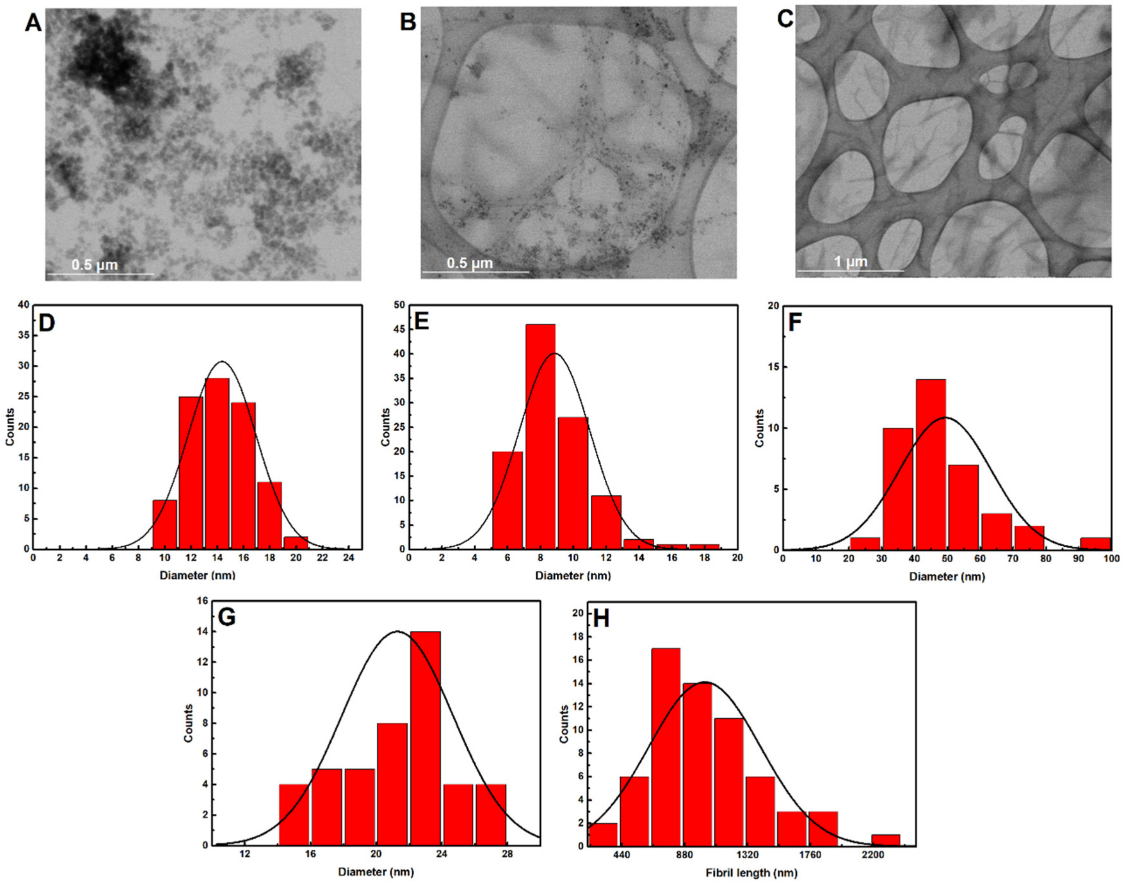

Figure S7. STEM images of (**A**) citrate-stabilized nanoparticles, (**B**) lipid-coated nanoparticles in the magnetogel, and (**C**) hydrogel used for the determination of size histograms. Size histograms of (**D**) citrate-stabilized and (**E**) lipid-coated nanoparticles, (**F**) thicker and (**G**) thinner fibril cross-section, and (**H**) fibril length obtained from STEM.

Magnetic hyperthermia heating efficiency

A phenomenological Box-Lucas equation was fitted to the temperature variation over time ($\Delta T(t)$) profiles during exposure to the alternating magnetic field. The equation is described by the parameter A (saturation temperature) and B (related with the curvature of the profile) as:

$$\Delta T(t) = A(1 - e^{-Bt}) \qquad \text{(S6)}$$

The product $A \times B$ at $t = 0$ is equivalent to the ratio $\Delta T/dt$ required to calculate SLP.

Table S6. Heating efficiency comparison for the nanoparticles in solution and in the magnetogels containing citrate-stabilized (CS) and lipid-coated (LC) nanoparticles at 1mg/mL. The ratio $\Delta T/dt$ was calculated through the Box-Lucas equation (SLP_f) and the difference of temperature change between the first 100 seconds (SLP_m).

System	f (kHz)	H (kA/m)	SLP_f (W/g)	SLP_m (W/g)	ILP_f (nHm^2/kg)	ILP_m (nHm^2/kg)
**CS Solution**	616.6	7.98	112.89	108.42	2.88	2.76
	382.6	12.76	106.56	100.46	1.71	1.61
	270.6	13.56	81.67	74.93	1.64	1.51
	161.6	13.56	43.42	39.35	1.46	1.32
**LC Solution**	616.6	10	6.57	5.02	0.17	0.13
	382.6	16	7.16	4.19	0.11	0.07
	270.6	17	4.87	3.77	0.10	0.08
	161.6	17	1.57*	1.67	0.05	0.06
**CS magnetogel**	616.6	10	24.96	23.02	0.64	0.59
	382.6	16	26.17	24.70	0.42	0.40
	270.6	17	19.27	18.42	0.39	0.37
	161.6	17	8.16	7.95	0.28	0.27
**LC magnetogel**	616.6	10	4.77	3.35	0.12	0.09
	382.6	16	6.55	3.77	0.11	0.06
	270.6	17	5.36	2.51	0.11	0.05
	161.6	17	3.59	0.84	0.12	0.03

*In the sample LC at the lowest frequency, the SLP was calculated using the difference between t = 300 s and t = 200 s due to the lag phase until 200 s.

Effect of nanoparticles on gel and doxorubicin fluorescence

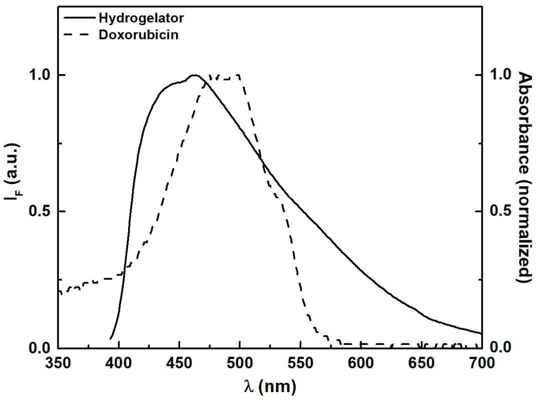

Figure S8. Normalized fluorescence emission spectrum (λ_{exc}= 375 nm) of the hydrogel and doxorubicin absorption spectrum.

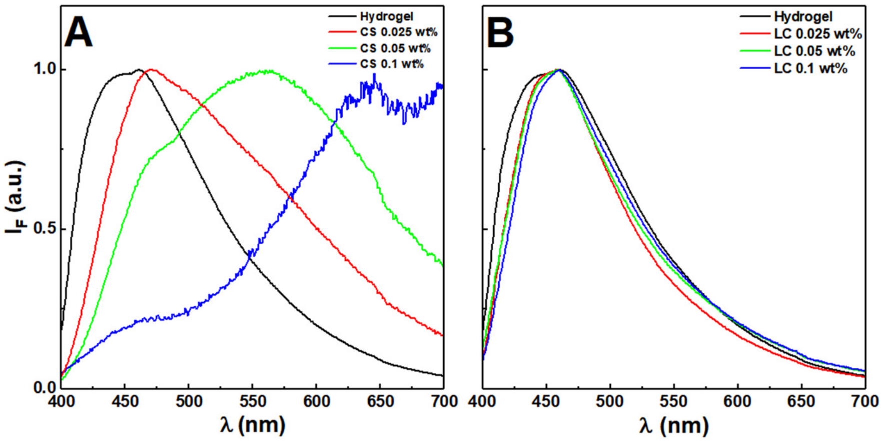

Figure S9. Normalized fluorescence emission spectra (λ_{exc}= 375 nm) of hydrogel and (A) magnetogels containing citrate-stabilized and (B) lipid-coated manganese ferrite nanoparticles at increasing nanoparticle concentration.

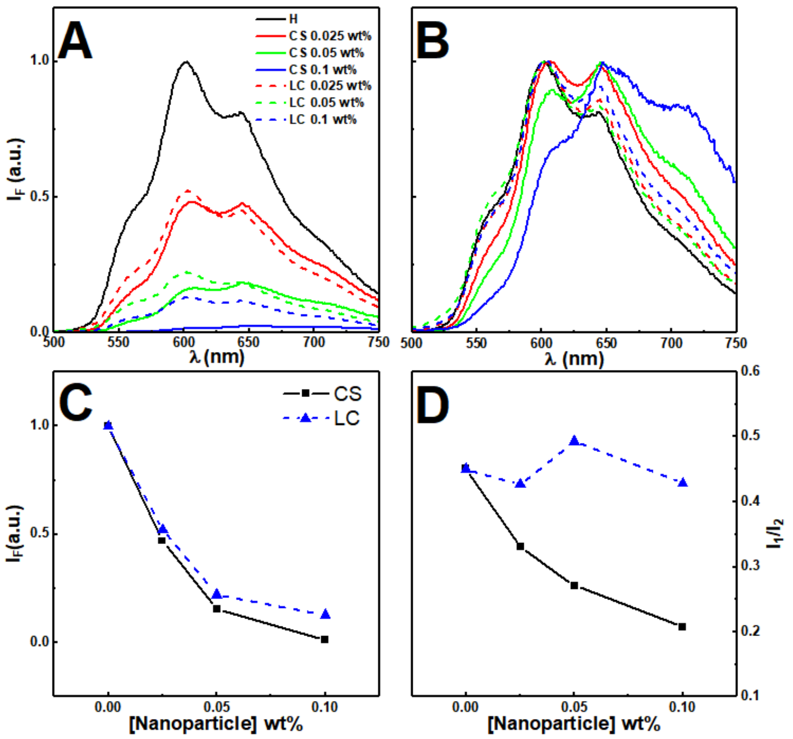

Figure S10. Fluorescence emission spectra (λ_{exc}= 480 nm) of (**A**) doxorubicin in the hydrogel and magnetogels containing citrate-stabilized and lipid-coated manganese ferrite nanoparticles at increasing nanoparticle concentration. (**B**) Normalized fluorescence emission spectra of doxorubicin in the respective systems. (C) Doxorubicin maximum fluorescence emission dependence on nanoparticle concentration. (D) Fluorescence emission I_1 (560 nm) to I_2 (600 nm) ratio dependence on nanoparticle concentration.

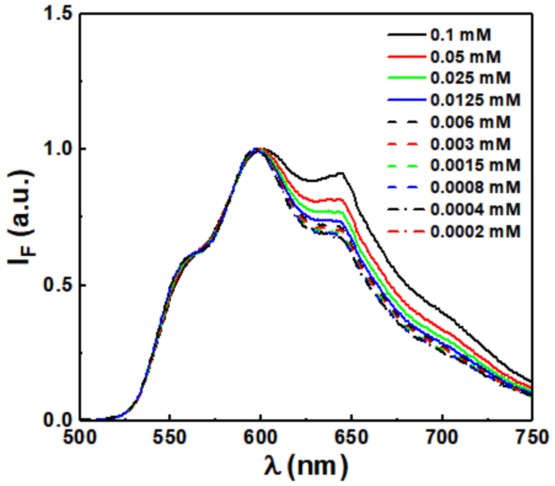

Figure S11. Normalized fluorescence emission spectra (λ_{exc}= 480 nm) of doxorubicin in pH=7 buffer. As the concentration is reduced, the shoulder around 645 nm decreases.

Doxorubicin release assays

Table S7. Coefficients of determination (R^2) obtained for doxorubicin release profiles (0.1 mM) in hydrogel and magnetogels containing lipid-coated (LC) and citrate-stabilized (CS) nanoparticles. Contact area of 0.78 cm^2.

System	Set	First-order	Hixson-Crowell	Higuchi	Korsmeyer-Peppas	Gompertz
**H**	Profile	0.94	0.93	0.87	0.96	0.98
	First 10 h	0.99	0.99	0.99	0.99	0.99
**CS**	Profile	0.87	0.78	0.94	0.95	0.97
	First 10 h	0.99	0.99	0.99	0.99	0.99
**LC**	Profile	0.82	0.72	0.85	0.89	0.95
	First 10 h	0.99	0.99	0.99	0.99	0.99

Table S8. Release coefficients of the Korsmeyer-Peppas and Gompertz model obtained for doxorubicin release profiles (0.1 mM) in hydrogel and magnetogels containing lipid-coated (LC) and citrate-stabilized (CS) nanoparticles. Contact area of 0.78 cm^2.

	Korsmeyer-Peppas		Gompertz		
**System**	K_S	n	X_{max}	a	b
**Hydrogel**	0.006	0.77	0.33	7.99	1.2
**LC**	0.007	0.63	0.17	6.14	1.3
**CS**	0.007	0.57	0.14	4.95	1.2
